# The Relation of Insanity to Civilisation.—III

**Published:** 1906-09-15

**Authors:** 


### THE RELATION OF INSANITY TO CIVILISATION.—III.

(*Continued from page* 412.)

### The Sane Turk.

Madness is rare in Turkey. This may be accounted for partly by the low degree of civilisation to which the country has attained, by the abstinence from fermented liquors, which the religion of Mahomet enjoins, and by the doctrine of predestination. The Turks are tormented by no religious scruples of metaphysical inquiries, by no misgivings concerning the future, and by no disappointment of their expectations. That which would drive an Englishman to despair elicits from them a sentiment of resignation only. The manners, education, and religion of the Turks are unfavourable to the development of the passions, and resignation is the distinctive feature of the national character.

If we quit the centre of civilisation and penetrate into the less civilised regions we find that the number of deranged persons, and of the causes of derangement, diminishes rapidly. In China mental alienation is of very infrequent occurrence. In the East Indies the forms of alienation are few; but religious madness prevails to a great extent, and the species of civilisation and the institutions that obtain are precisely those which would give it predominance.

It may perhaps be said that cases of insanity are more frequent in number and more varied in form among nations which are civilised than among those which are less enlightened. Among the former mental derangement is chiefly owing to the action of moral causes, while among the latter physical causes are the most influential in producing the malady. Each age, each country, witnesses the outbreak of species of insanity determined by the influence of ideas then or there predominant; each species, therefore, bearing as it were the impress of the epoch. Each remarkable event, each great public calamity, increases the number of insane persons. The proportion of insane persons to the whole population is most considerable among the nations that have attained a high degree of civilisation.

### The Era of Hospitals.

The era of the real hospitals for the insane properly belongs to the nineteenth century. There had been establishments here and there in different parts of the world, it is true, certain asylums or places of restraint before the beginning of the nineteenth century: we find mention in history of such a place established by monks at Jerusalem in the latter part of the fifth century. There is evidences that even earlier than this, in Egypt and Greece, the insane were treated as individuals suffering from disease. Until as late as the middle of the eighteenth century mildly insane persons were cared for at shrines, or wandered homeless about the country. Such as were deemed a menace to the community were sent to ordinary prisons or chained in dungeons. Thus large numbers of lunatics accumulated in the prisons, and slowly there grew up a sort of distinction between them and criminals, which at length resulted in a separation of the two classes.

Twenty-five years after Pinel had, in 1792, struck the chains from the lunatics huddled in the Salpêtrière, and Bicêtre of Paris, and called upon the world to realise the horrible injustice done to this wretched and suffering class of humanity, Esquirol wrote of the insane in France and all Europe: "These unfortunate people are treated worse than criminals, reduced to a condition worse than animals. I have seen them naked, covered with rags, and having only straw to protect them against the cold moisture and the hard stones they lie upon; deprived of air, of water to quench thirst, and all the necessaries of life; given up to men gaolers, and left to their surveillance. I have seen them in their narrow and filthy cells, without light and air, fastened with chains in these dens in which one would not keep wild beasts. This I have seen in France, and the insane are everywhere in Europe treated in the same way." It was not until 1838 that the insane in France were all transferred from small houses of detention, workhouses, and prisons to asylums specially constructed for this purpose.

### Great Advance in the Care of the Insane.

These facts show that no great advance in the humane and scientific care of the insane was made till towards the middle of the nineteenth century. Only then did the actual metamorphosis of asylums for detention into hospitals for treatment begin to take place. Hand in hand with this progress there has grown, and still is growing, a tendency to subdivision and specialisation of hospitals for this purpose. The statistics of insanity have nowadays been fairly well established. The ratio of insane to normal population is about 1 to 300 among civilised peoples. This proportion varies within narrow limits in different races and countries. It is probable that intemperance in the use of alcohol and drugs, the spread of venereal diseases, and the over-stimulation in many directions induced by modern social conditions have caused an increase of insanity as compared with past centuries.

### Inherited Predisposition.

Certain individuals inherit from their ancestors the predisposition to insanity, and this can often be recognised by the habits, manners, feeling, and tastes, and mode of thinking in certain individuals. They are predestined to madness. The intermarriage of such individuals or the marriage of an individual inheriting such a neuropathic taint with another person of sickly constitution or disposed to phthisis may transmit to their offspring many miseries. Dr. Maudsley's remarks on the subject of marriages are so sensible that they are deserving of being quoted :—

"When one considers the reckless way in which persons, whatever the defects of their mental and bodily constitution, often get married without sense of responsibility for the miseries which they entail upon those who will be the heirs of their infirmities, without regard in fact to anything but their own present gratification, one is driven to think either that man is not the pre-eminently reasoning and moral animal which he claims to be, or that there is in him an instinct which is deeper than knowledge. He has persuaded himself, rightly or wrongly, that in his case there is in the feeling of love between the sexes something of so sacred and mysterious a character as to justify disregard to consequences in marriage. We have only to look at the large part which love fills in novels, poetry, and painting, and to consider what a justification of unreason in life it is held to be, to realise what a hold it has on him in his present state of development, and what a repugnance there would be to quench its glow by cold words of reason. At bottom, however, there is nothing particularly holy about it; on the contrary, it is a passion which man shares with other animals."

### Credit to Latterday Civilisation.

The modern hospital for the insane does credit to latterday civilisation. Progress in asylum architecture has advanced more slowly in countries where monasteries and cloisters abounded than in countries where fixed models did not exist. Architects have had a freer hand in America,

Australia, and Germany, and even in Great Britain, than in the Catholic countries of Europe. At the present time Germany approaches nearest to an ideal standard of provision for the insane. The highest and best idea which has yet been attained is that of small hospitals for the acutely insane in all cities of more than 50,000 inhabitants, and of colonies for the chronic insane in the rural districts adjacent to centres of population. Every university town in Germany has its psychopathic hospital, and in the matter of colonies for the insane many of the provinces have already established them. Among the colonies, that at Alt Scherlitz, near Leipsic, is the oldest and most successful, and is pre-eminent in its close approach to the ideal village or colony system. Professor Kraeplin, of Heidelberg ("Psychiatrie," 6th Edit., 1899), states that the effort is made everywhere in Germany to give the exterior of asylums, by segregation of the patients in separate home-like villas, rather the appearance of hamlets for working people than prisons for the insane, and says further, that the whole question of the care of the insane has found solution in the colony system, which is the best and cheapest method of support.

